# BMP-12 Treatment of Adult Mesenchymal Stem Cells *In Vitro* Augments Tendon-Like Tissue Formation and Defect Repair *In Vivo*

**DOI:** 10.1371/journal.pone.0017531

**Published:** 2011-03-11

**Authors:** Jonathan Y. Lee, Zuping Zhou, Peter J. Taub, Melissa Ramcharan, Yonghui Li, Takintope Akinbiyi, Edward R. Maharam, Daniel J. Leong, Damien M. Laudier, Takuya Ruike, Phillip J. Torina, Mone Zaidi, Robert J. Majeska, Mitchell B. Schaffler, Evan L. Flatow, Hui B. Sun

**Affiliations:** 1 Leni and Peter W. May Department of Orthopaedics, Mount Sinai School of Medicine, New York, New York, United States of America; 2 Division of Plastic Surgery, Mount Sinai School of Medicine, New York, New York, United States of America; 3 Department of Biomedical Engineering, City College of New York, New York, New York, United States of America; 4 The Mount Sinai Bone Program, Mount Sinai School of Medicine, New York, New York, United States of America; Ohio State University, United States of America

## Abstract

We characterized the differentiation of rat bone marrow-derived mesenchymal stem cells (BM-MSCs) into tenocyte-like cells in response to bone morphogenetic protein-12 (BMP-12). BM-MSCs were prepared from Sprague-Dawley rats and cultured as monolayers. Recombinant BMP-12 treatment (10 ng/ml) of BM-MSCs for 12 hours *in vitro* markedly increased expression of the tenocyte lineage markers scleraxis (Scx) and tenomodulin (Tnmd) over 14 days. Treatment with BMP-12 for a further 12-hour period had no additional effect. Colony formation assays revealed that ∼80% of treated cells and their progeny were Scx- and Tnmd-positive. BM-MSCs seeded in collagen scaffolds and similarly treated with a single dose of BMP-12 also expressed high levels of Scx and Tnmd, as well as type I collagen and tenascin-c. Furthermore, when the treated BM-MSC-seeded scaffolds were implanted into surgically created tendon defects *in vivo*, robust formation of tendon-like tissue was observed after 21 days as evidenced by increased cell number, elongation and alignment along the tensile axis, greater matrix deposition and the elevated expression of tendon markers. These results indicate that brief stimulation with BMP-12 *in vitro* is sufficient to induce BM-MSC differentiation into tenocytes, and that this phenotype is sustained *in vivo*. This strategy of pretreating BM-MSCs with BMP-12 prior to *in vivo* transplantation may be useful in MSC-based tendon reconstruction or tissue engineering.

## Introduction

Tendon injuries are common in adults, necessitating over 300,000 surgical tendon repairs each year in the United States [1]. Unfortunately, tendons heal poorly due to their limited regenerative potential, and many repairs require revision [2]. Moreover, recovery following tendon repair can be protracted, ranging from months to years, and at best, healed tendons possess 60% of their initial mechanical properties [3]. Recent tissue regeneration strategies aim to improve the outcome of tendon repair. Some of these approaches utilize adult mesenchymal stem cells (MSCs) to form new tendon tissues [4]. Adult MSCs are favored for tissue engineering because of their ease of isolation, rapid propagation, multilineage differentiation capabilities and low immunogenicity, among other considerations [5].

Several studies have demonstrated MSC differentiation into tenocyte-like cells in response to chemical factors including bone morphogenetic proteins (BMPs), transforming growth factor-β (TGF-β), and fibroblast growth factor (FGF) [6], [7], [8], [9], [10]. BMPs, members of the TGF-β/BMP superfamily with important regulatory roles in the development and morphogenesis of multiple organs and tissues [11], [12], [13], [14], are of particular interest with respect to tendon differentiation. BMPs 7, 12, 13 and 14 have been implicated in the neoformation and repair of tendons [8], [15], and of these, BMP-12, the human homologue of mouse growth and differentiation factor 7 (GDF-7), has been shown to promote tendon differentiation and formation both *in vivo* [16] and *in vitro* [7], [17].

Collectively, these studies highlight a teno-inductive capacity of BMP-12 that may be exploited therapeutically for tendon repair. A major concern surrounding the use of BMP-12 *in vivo*, however, is that this cytokine can also affect the differentiation of other cell types including muscle [18], cells of secretary glands in the male reproductive system [19] and several neuronal cell lineages [20], [21], [22]. In the present study, we characterized tenocytic differentiation of rat bone marrow-derived MSCs (BM-MSCs) treated with BMP-12 *in vitro*, and tested whether the tenocyte-like phenotype would be sustained following implantation in an *in vivo* model of tendon damage.

## Materials and Methods

### Isolation and culture of MSCs

Sprague-Dawley female rats (5–6 month old) were purchased from Charles River Laboratories (Wilmington, MA). The animals were housed in a standard animal facility at Mount Sinai School of Medicine and all experiments involving animal use were performed in accordance with the Institutional Animal Care and Use Committee. BM-MSCs were prepared as described [23]. Briefly, bone marrow was collected by flushing femur and tibia with medium and single cell suspensions prepared by repetitively pipetting through 18-gauge needles. After centrifugation, cell pellets were resuspended in growth medium consisting of Dulbecco's modified eagle medium (DMEM, Invitrogen), 10% fetal bovine serum (FBS, Gibco), 100 U/ml penicillin, and 100 mg/ml streptomycin, (1% P/S, Invitrogen), then seeded and incubated in complete medium. The medium was replaced 24 hours later, and every 2 to 3 days thereafter during a total 2 week culture, after which cells were detached by trypsinization and replated for experiments.

BM-MSCs were cultured in growth medium to approximately 80∼90% confluence, then starved for 12 hrs in DMEM supplemented with 1% FBS followed by treatment with 10 ng/ml recombinant BMP-12 (R&D Systems) in the same low-serum medium for either 12 or 24 hours. After treatments, BM-MSCs were cultured in growth medium in the absence of BMP-12 for extended periods.

### Single CFU-F assay

BM-MSCs at passage 1 were plated at a density of 2×10^2^/35 mm dish and treated with different concentrations of BMP-12 for 12 h (1-hit) or 12 h+12 h, as described. After 14 days, media was removed and the cultures were immunostained for Scx and Tnmd using anti-Scx (Abcam) and anti-Tnmd (Santa Cruz), respectively, followed by anti-rabbit secondary antibody (Dako; code no. K1015) and chromagenic detection. Negative controls were prepared using irrelevant isotype matched primary antibodies (Dako; code no. X931 or X0936) in place of authentic primary antibody. Total, Scx-positive and Tnmd-positive colonies were counted microscopically at 40X magnification by a phase contract microscope. Colonies containing more than 50 cells were scored.

### Differentiation of cells in scaffolds

BM-MSCs were suspended in growth medium at 1×10^6^ cells/mL and 0.25 mL was seeded onto sterilized 5 mm×2 mm collagen sponge scaffolds (Zimmer Dental). Cell-seeded scaffolds were placed in culture dishes and incubated for 2 hours in a minimum volume of growth medium, after which more medium was applied to submerge the scaffolds. After an additional 24-hour culture, cells seeded in scaffolds were treated with 10 ng/mL of recombinant BMP-12 for 12 hours. The medium was then replaced with fresh growth medium and scaffolds were either cultured for an additional 7 days or immediately implanted into partial calcaneal tendon defects in rats.

### *In vivo* implantation

Animals were anesthetized with isoflurane gas (2–3% by volume, 0.4 L/min) and the skin overlying the left calcaneal tendon was shaved and sterilized with alcohol and betadine pads. A longitudinal incision was made in the left hindlimb to expose the calcaneal tendon, and a 5 mm long, half-width partial defect was created on the lateral border of the tendon. A scaffold that was either (1) unseeded [control, n = 8], (2) seeded with BM-MSCs without BMP-12 treatment [n = 8], or (3) seeded with BMP-12 treated BM-MSCs [n = 8] was sutured into the defect using 10-0 nylon suture (Ethicon). The skin incision was then closed with 4-0 vicryl (Ethicon) and the animals were returned to their cages and allowed to resume normal activity. Buprenorphine (0.015 mg/kg) was administered subcutaneously for post-operative pain analgesia. During the 3-week experimental period, no infections, animal deaths or body weight losses were observed.

### Histological analysis

Scaffolds cultured *in vitro* and dissected calcaneal tendon tissues were fixed in 10% neutral buffered zinc-formalin and embedded in polymethyl methacrylate [22]. For standard histological evaluation, sections were stained with methylene blue, hematoxylin and eosin (H&E), Masson's Trichrome, or toluidine blue. For immunohistochemical staining, sections were incubated overnight at 4°C with either anti-Scx (1∶150, Abcam), anti-Tnmd (1∶150, Santa Cruz) or nonimmune antiserum (1∶150, negative control), followed by a 30 minute incubation with a secondary antibody conjugated with horseradish peroxidase [anti-rabbit (1∶1000, Santa Cruz)] in 0.1% TBST. Sections were counterstained with 0.1% methylene blue and image analysis was carried out with Axiovision v4.6 software (Zeiss).

### Quantitative PCR

Total RNA was extracted from seeded scaffolds cultured *in vitro* and flash-frozen, pulverized implants from the left calcaneal tendons using the QIAshredder (Qiagen, Valencia, CA) and RNeasy Minikit (Qiagen) according to manufacturer's instructions. Isolated RNA was reverse transcribed with Super Script II reverse-transcriptase and Oligo(dT)_12–18_ primers (Invitrogen) and the cDNA was subjected to real-time PCR with SYBR Green PCR Master Mix (Applied Biosystems). The primers used for PCR were Scx (NM_001130508 - f: ctggcctccagctacatttc, r: ccgtctttctgtcacggtct); Tnmd (NM_022290 - f: ccagacaagcaagcgagga, r: aacttcctattagactctcc); Tn-C (U15550 – f: cagaagccttggccatgtg, r: gcactctctcccctgtgtagga); Col Iα1 (Z78279 – f: ggagagtactggatcgaccctaac, r: ctgacctgtctccatgttgca); GAPDH (BC059110 - f: acagcaacagggtggtggac, r: tttgagggtgcagcgaactt); β-actin (NM_031144 – f: cacccgcgagtacaaccttc, r: cccatacccaccatcacacc). GAPDH and β-actin levels were used as internal controls. Differences in mRNA levels were assessed by one-way ANOVA followed by post-hoc Tukey test using StatView v5.0 (SAS Institute). p≤0.05 was considered significant.

## Results

### BMP-12 stimulates the expression of Scx and Tnmd in rat BM-MSC cultures

**Figure 1:**
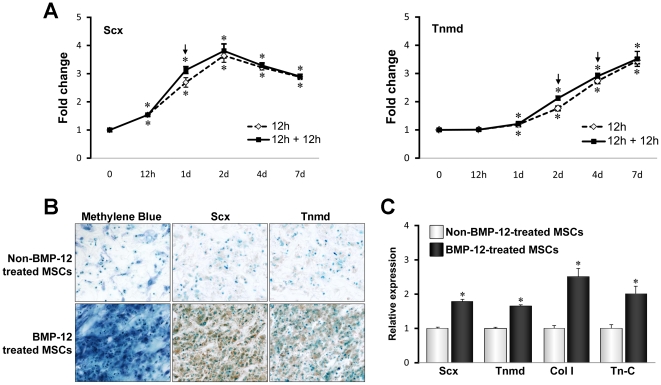
Effects of BMP-12 on the rat BM-MSC differentiation toward tenocyte lineage *in vitro*. (**A**) BMP-12-induced expression of Scx and Tnmd in monolayer cultures. Rat BM-MSCs at passage 1 were plated, and 24 hours later were treated with 10 ng/ml of BMP12 for 12 hours (“1-hit”) or for 24 hours (12 hours plus another 12 hours). Following BMP-12 stimulation, cells were cultured in the absence of BMP-12 for the indicated times. mRNA levels were determined by qRT-PCR. Data are expressed as mean ± S.D. (*n* = 3). * represents p<0.05. (**B**, **C**) BMP-12-mediated tenocytic differentiation of rat BM-MSC in collagen scaffolds. BM-MSCs (2.5×10^5^ cells) were seeded onto sterilized 5 mm×2 mm collagen sponge scaffolds and incubated in growth media. After 24-hour culture, cells were left untreated or treated with 10 ng/mL of BMP-12 for 12 hours. The media was then replaced with fresh growth medium and the scaffolds were cultured in the absence of BMP-12 for 14 days. At the end of culture, cells were stained with methylene blue (B, left panels, 20X magnification) or subjected to immunohistochemical staining for analysis of Scx and Tnmd protein (Middle and right panels, 20X magnification). Cells were also lysed with Trizol and gene expression was determined by qRT-PCR (C). Data shown in (B) are representative of 3 independent experiments. Data in (C) are expressed as mean ± S.D. (*n* = 3). * represents p<0.05.

Scx and Tnmd are two genes predominantly expressed in tendons, and are considered the most reliable phenotypic markers of the tenocytic lineage. To test the feasibility of using BMP-12 as an inducer of BM-MSC tenocyte differentiation, we first compared the effects of a single 12 hour treatment and 2 sequential 12 hour treatments with 10 ng/ml BMP-12 on the expression of Scx and Tnmd in rat BM-MSC monolayer cultures. Following BMP-12 treatment, all cells were cultured in the absence of BMP-12, and then collected at different times for gene expression analysis. qPCR showed that a single 12 or 24 hour exposure to BMP-12 enhanced expression of both Scx and Tnmd. Scx mRNA expression was highest on day 2 and decreased thereafter, but still remained above basal levels (Figure 1A, left panel). Tnmd expression lagged behind Scx, and increased continuously over the 7-day culture period (Fig. 1A, right panel). Interestingly, no differences in Scx and Tnmd expression were observed between single and double BMP-12 treatments (Fig. 1A).

Similar results were obtained when tenocyte differentiation was assessed by CFU assays. Fourteen days after either single or double BMP-12 treatment, over 80% of colonies expressed both Scx and Tnmd (Table 1). These results indicate that once induced, Scx and Tnmd expression remain elevated for an extended period of time in the absence of BMP-12.

**Table 1:** Effect of BMP-12 on Scx and Tnmd protein expression in colonies derived from rat BM-MSCs.

Gene	Treatment Duration	Positive colonies per well	Total colonies per well	% Positive colonies
Scx	0 h	2.5±1.3	66.8±5.2	3.74
	12 h	58.8±4.0*	67.8±4.4	86.72
	12 h+12 h	60.0±5.2*	68.4±4.6	87.72
Tnmd	0 h	2.3±1.3	68.5±6.9	3.36
	12 h	59.0±3.2*	68.8±3.0	85.75
	12 h+12 h	57.4±3.9*	68.4±5.6	84.16

Colony forming assays were performed on untreated cells, or on cells treated with BMP-12 for 12 h (“1-hit”) or for 12 h + 12 h. Total, Scx-positive and Tnmd-positive colonies were counted on Day 14 after plating. Data are presented as mean ± S.D. (n = 6); * represents P<0.05 for BMP-12 treated cells compared to untreated controls.

### BMP-12 accelerates BM-MSC tenocyte differentiation in 3D collagen scaffolds *in vitro*

We next explored the effect of BMP-12 treatment on the differentiation of BM-MSCs-seeded in three-dimensional collagen sponge scaffolds. Tenocyte differentiation was evaluated based on cellular morphology and organization, as well as the expression of tenocyte-lineage marker genes. Methylene blue staining showed that BM-MSCs treated with BMP-12 after seeding in collagen scaffolds displayed increased cell numbers and elongation compared with untreated cells after 7 days of *in vitro* incubation (Fig. 1B, left panels). In addition to these histological changes, enhanced staining of Scx and Tnmd proteins was observed in seeded-BMP-12-treated samples, but not in those without BMP-12 treatment (Fig. 1B, middle and right panels). Finally, mRNA levels of Scx, Tnmd, and two other genes expressed by tendon cells, type I collagen (Col I) and tenascin-C (Tn-C) were increased in BMP-12-treated cells (Fig. 1C).

### BMP-12-treated BM-MSCs augment tendon-like tissue formation in calcaneal tendon defects *in vivo*

**Figure 2:**
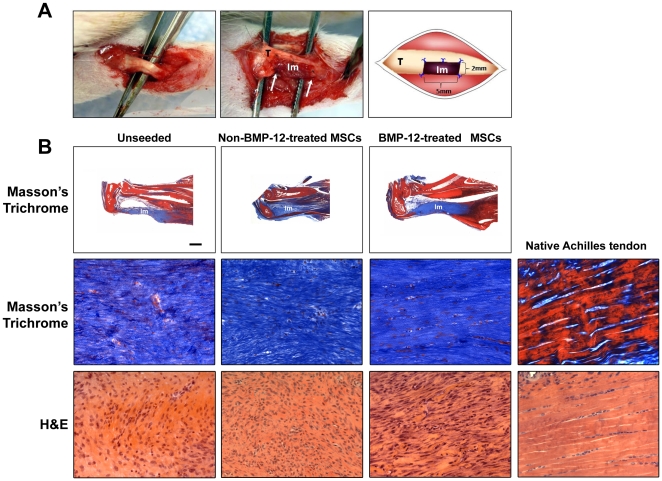
Fomation of tendon-like tissues by BMP-12-treated rat BM-MSCs in calcaneal tendon defects *in vivo*. (**A**) Exposed rat calcaneal tendon of the left hind-limb (Left panel). Scaffolds were implanted into half-width, 5 mm-long partial calcaneal tendon defects using 10-0 nylon (Middle panel). A schematic drawing highlights the spatial relationship between the tendon and the implant (Right panel). “T” denotes tendon; “Im” denotes implant; arrows denote the tendon-implant interface. Note that 5 interrupted 10-0 nylon sutures were used to secure the implant in the defect and that the implant completely filled in the 5 mm×2 mm calcaneal tendon defect. (**B**) Cells were cultured and treated as described for Fig. 1B, C. Scaffolds with or without cell seeding were implanted as in Fig. 2A. After 3 weeks, implants and Achilles tendons from naïve animals were dissected and subjected to histological analysis (Masson's Trichrome or H&E staining). Seeded BMP-12-treated implants but not unseeded and non-BMP-12-treated group exhibited higher cellularity, increased formation of collagen, and organized fibrous structures, indicating robust formation of tendon-like tissues. 20X magnification.

**Figure 3:**
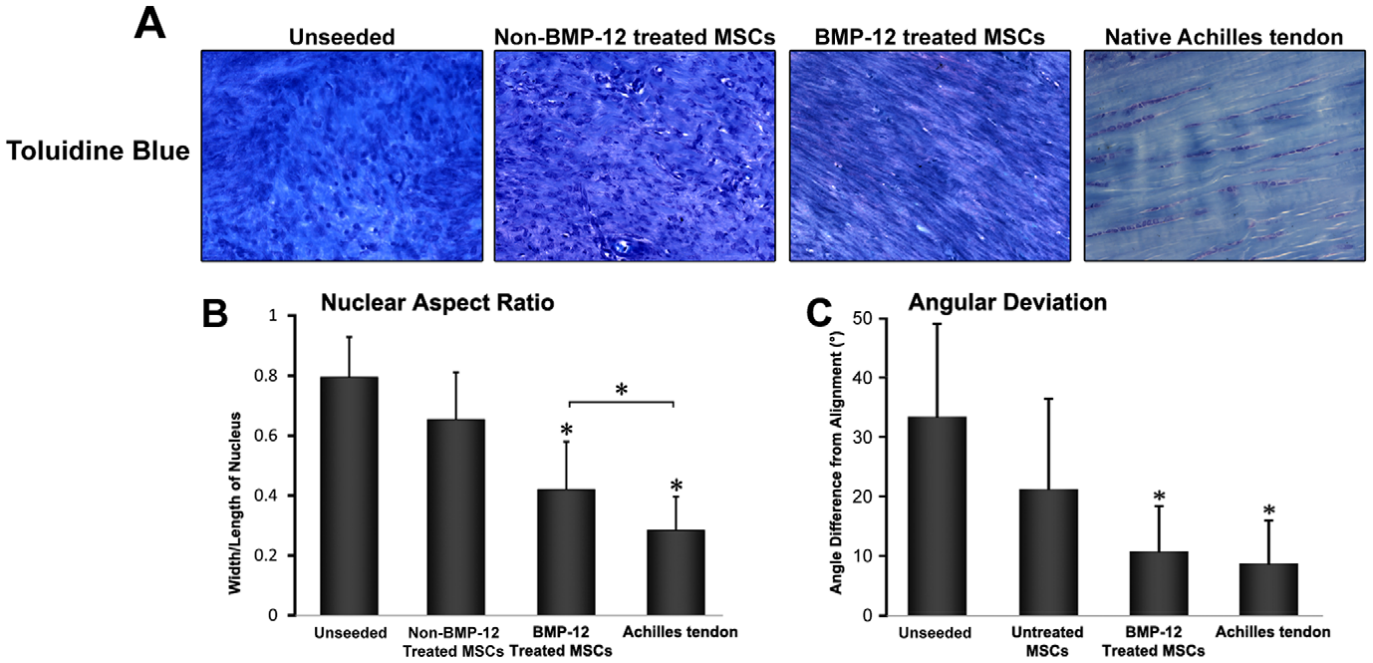
Enhanced cell alignment following BMP-12-treatment of scaffolds seeded with BM-MSCs. Rat BM-MSCs were cultured and implanted as in Fig. 2. (**A**) Toluidine blue staining (20X magnification) revealed increased cell elongation and cellular alignment/organization, within the BMP-12-treated BM-MSCs implants. (**B**) Nuclear aspect ratio (width *vs* length of nucleus), and (**C**) Angular deviation (angle between individual nuclear axis and longitudinal axis based on general alignment). A smaller value of nuclear aspect ratio and nuclear orientation angle indicated greater cellular elongation and alignment in cells treated with BMP-12, as compared to untreated cells. * represents p<0.05.

Having shown that BMP-12-treated BM-MSCs acquired several phenotypic properties of tenocytes *in vitro*, we next tested whether these changes would also be observed *in vivo*. For this purpose, BM-MSCs seeded in collagen scaffolds treated *in vitro* with either BMP-12 or vehicle were implanted into surgically created defects (5 mm×2 mm) in calcaneal tendons (Fig. 2A). Unseeded scaffolds were also implanted as additional controls. Three weeks after implantation, animals were sacrificed and the implants were recovered for morphological and gene expression analysis. A histological analysis demonstrated that all implants, including those not seeded with BM-MSCs, contained abundant numbers of cells, (Figs. 2B and 3A), and that all the implants showed qualitative evidence of vascularization. However, the organization of both cells (visualized mainly by H&E staining) and matrix (seen by H&E, Masson Trichrome and toluidine blue) was most pronounced in the implants where cells had been treated with BMP-12. The BMP-12-treated cells were largely spindle-shaped and like the fibrous matrix, were generally well aligned/organized along the longitudinal (tensile) axis of the tissue (Fig. 2B, Fig. 3A). By contrast, the specimens from the non-BMP-12-treated and unseeded groups exhibited minimal development of tendon-like morphology, and instead remained poorly organized (Fig. 2B, Fig. 3A). We found that the nuclei of BMP-12-treated cells exhibited morphological features resembling the organization of cells in native tendons, while untreated MSCs did not. The BMP-12-treated/seeded group had a slightly higher value of both nuclear aspect ratio (width *vs* length of nucleus) (Fig. 3B) and value of nuclear orientation angle (angle of nuclear axis from tissue/cell longitudinal axis or general alignment) than the naïve group, but those values were significantly lower as compared to the control groups (Figs. 3B, C).

**Figure 4:**
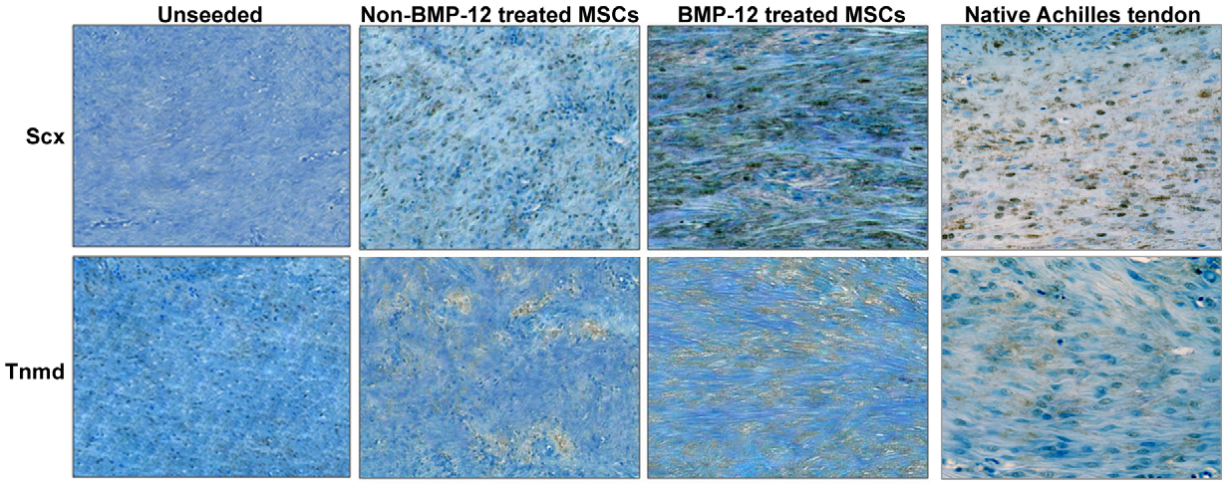
Increased expression of Scx and Tnmd proteins in seeded BMP-12-treated implants. Implants as in Fig. 2 and naïve Achilles tendons were dissected and tissue sections were immunohistochemically stained with specific anti-Scx or anti-Tnmd antibodies as described in Material and Methods. Data shown are representative of three independent experiments (20X magnification).

**Figure 5:**
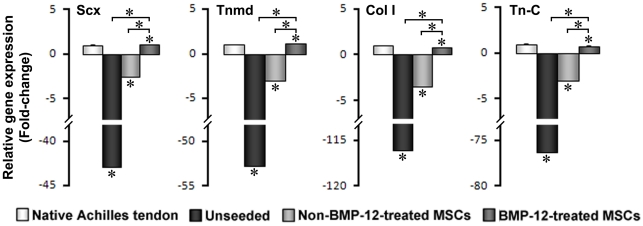
Induction of tendon cell-related genes by BMP-12 in rat BM-MSC implants in calcaneal tendon *in vivo*. Samples were collected as described in Methods and Materials. mRNA levels were determined by qRT-PCR and normalized against samples of the non-BMP-12-treated group. The gene expression of all experimental groups was determined as fold changes relative to native tendon samples. While unseeded implants expressed minimal Scx, Tnmd, Col I, and Tn-C, BMP-12-treated implants had significantly increased expression of these genes compared to non-BMP-12-treated implants. Data are expressed as mean ± S.D. (n = 3). * represents p<0.05.

Immunohistochemical analysis showed greatly enhanced staining of Scx and Tnmd proteins in sections prepared from seeded BMP-12-treated implants compared to unseeded or non-BMP-12-treated groups (Fig. 4). The expression levels of both Scx and Tnmd in BMP-12-treated scaffold implants were even higher than in the naïve tendons (Fig. 4). Consistently, RT-PCR analysis revealed that cells from the seeded/BMP-12-treated implants expressed much higher mRNA levels of Scx, Tnmd, Col I, and Tn-C than control samples (Fig. 5). These differences *in vivo* were even greater than those observed in 3D cultures *in vitro*. Taken together, these data suggest that rat BM-MSCs pretreated with BMP-12 have acquired full tenogenic capability, thus being able to form tendon-like tissues after implantation *in vivo*.

## Discussion

The results of this study show that BMP-12 is a highly effective inducer of tenocyte-like cell differentiation in BM-MSCs, and that the phenotype induced by BMP-12 appears to be sustained both *in vitro* and *in vivo* without the need for further exposure to exogenous BMP-12. The basis for this persistent BMP effect is so far unclear. It seems unlikely that BMP-12 induces its own synthesis in BM-MSCs; rather, BMPs more often may downregulate their own expression or induce production of antagonists [24]. Perhaps direct cellular responses to BMP-12 induction are intrinsically long-lived, possibly reflecting changes (e.g. in chromatin organization) that are characteristic of a new state of differentiation [25]. These findings suggest that use of BMP-12 to “pre-differentiate” tenocytes from stem cell populations seeded into collagen scaffolds *in vitro* could be an effective approach to engineer artificial tissues for tendon repair.

Because no single phenotypic marker is known to identify mature tenocytes conclusively, we utilized a combination of genetic and morphologic traits to assess tenocytic differentiation in response to BMP-12. Scx is a transcriptional regulator first expressed in progenitors of tendon cells [6], while the transmembrane protein Tnmd is expressed by more mature tenocytes and has been implicated in regulating their proliferation and matrix organization [26]. Further study found that expression of Scx is essential for subsequent expression of Tnmd [27]. Consequently, the sequential expression of Scx and Tnmd in this study is consistent with progressive differentiation of these cells along a single, likely tenocytic pathway. Moreover, the expression pattern of Scx and Tnmd in response to BMP-12 was linked to changes in cellular morphology (elongation) and overall cellular organization (increasing side-by-side alignment within collagen scaffolds) similar to the tendon-like tissues produced in other systems in response to morphogens or mechanical stimuli [8], [28].

BM-MSCs are multipotential and can be induced to differentiate into a range of cell types including osteoblasts, chondrocytes and adipocytes [4]. The high percentage of BM-MSC colonies expressing tenocytic markers in response to BMP-12 induction (over 85% were positive for Scx and Tnmd) not only demonstrates its effectiveness as an inducer of tenocyte differentiation, but also indicates that the tenocyte pathway of differentiation is preferred under the conditions of this experiment. Furthermore, the finding that a single dose of BMP-12 was sufficient to induce BM-MSC differentiation is consistent with the activities of other BMP family members like BMP-2, where single, short-term administration is highly effective in cartilage and bone induction [29], [30].

Not only do BM-MSCs exhibit multipotentiality, mature cells of mesenchymal lineage exhibit a degree of phenotypic plasticity, and can dedifferentiate and redifferentiate along alternate pathways [31]. The phenotypic stability of tenocytes following *in vitro* induction by BMP-12 is of particular interest for tissue engineering purposes. The fact that phenotypically mature cells like tenocytes can dedifferentiate over time raises the possibility that engineered tissues formed from those cells could not be sustained *in vivo*. While the long-term *in vivo* phenotypic stability of BM-MSCs induced *in vitro* with BMP-12 has yet to be fully evaluated, our results demonstrating sustained cell numbers and tissue organization over 3 weeks of *in vivo* implantation, without repeated BMP-12 treatments suggest that this approach may be useful and merits more extensive investigation. Moreover, the use of BMP-12 *in vitro* to induce tenocytic differentiation avoids concerns about the introduction *in vivo* of large amounts of BMP-12 protein, vectors carrying the BMP-12 gene, or cells transfected or transduced to overexpress the BMP-12 gene.

Taken together, our study has demonstrated that rat BM-MSCs treated with BMP-12 express high levels of tendon-related genes, undergo differentiation into tenocyte-like cells, and efficiently form tendon-like tissues *in vivo*. Therefore, we conclude that a brief treatment of rat BM-MSCs prior to implantation is sufficient to induce differentiation toward tenocytic lineage. These data could be useful in helping develop BM-MSC-based tendon tissue engineering approaches for tendon repair.
